# Cost effectiveness of OptiMal^® ^rapid diagnostic test for malaria in remote areas of the Amazon Region, Brazil

**DOI:** 10.1186/1475-2875-9-277

**Published:** 2010-10-11

**Authors:** Maria Regina Fernandes de Oliveira, Almério de Castro Gomes, Cristiana M Toscano

**Affiliations:** 1 School of Medicine, University of Brasília, Brasilia, Brazil; 2 School of Public Health, University of São Paulo, São Paulo, Brazil; 3 Department of Community Health, Federal University of Goiás, Goiânia, Brazil

## Abstract

**Background:**

In areas with limited structure in place for microscopy diagnosis, rapid diagnostic tests (RDT) have been demonstrated to be effective.

**Method:**

The cost-effectiveness of the Optimal^® ^and thick smear microscopy was estimated and compared. Data were collected on remote areas of 12 municipalities in the Brazilian Amazon. Data sources included the National Malaria Control Programme of the Ministry of Health, the National Healthcare System reimbursement table, hospitalization records, primary data collected from the municipalities, and scientific literature. The perspective was that of the Brazilian public health system, the analytical horizon was from the start of fever until the diagnostic results provided to patient and the temporal reference was that of year 2006. The results were expressed in costs per adequately diagnosed cases in 2006 U.S. dollars. Sensitivity analysis was performed considering key model parameters.

**Results:**

In the case base scenario, considering 92% and 95% sensitivity for thick smear microscopy to *Plasmodium falciparum *and *Plasmodium vivax*, respectively, and 100% specificity for both species, thick smear microscopy is more costly and more effective, with an incremental cost estimated at US$549.9 per adequately diagnosed case. In sensitivity analysis, when sensitivity and specificity of microscopy for *P. vivax *were 0.90 and 0.98, respectively, and when its sensitivity for *P. falciparum *was 0.83, the RDT was more cost-effective than microscopy.

**Conclusion:**

Microscopy is more cost-effective than OptiMal^® ^in these remote areas if high accuracy of microscopy is maintained in the field. Decision regarding use of rapid tests for diagnosis of malaria in these areas depends on current microscopy accuracy in the field.

## Background

In Brazil, 99.8% of malaria cases occur in the Amazon Region, of which more than 70% are due to *Plasmodium vivax *[1]. Risk for malaria is given by the malaria annual parasitic incidence (API) which stratifies in high (> 49.9 malaria cases/1,000 population), medium (10 - 49.9 cases/1,000 population) or low (< 10 cases/1,000 population) risk [2,3]. Between 2003 and 2007, the API of the Amazon Region ranged from 18.3 to 26.6 cases/1,000 population. In 2007, 457,659 cases were registered in the region, with an API of 19.2 cases/1,000 population [1].

Early and accurate diagnosis is crucial as it enables the institution of prompt and adequate treatment, minimizing the risk of complications, the duration of disease and therefore its medical and economic burden. Reliable and efficient diagnostic methods are therefore essential in endemic countries [4].

Microscopy with the thick smear method is the most common method for malaria diagnosis. It is of low cost [5], and allows direct visualization of the parasite, therefore, identifying all of the *Plasmodium *species and quantifying parasites in the blood. Its execution requires well-maintained laboratories, and experienced and highly trained professionals. The technique has its inherent limitations, such as variability in the quality of the blood smear, inability to determine the parasite species when parasitaemia is very low and loss of slide quality over time. Moreover, the procedure to prepare and read slides may vary among technicians [5-8].

In Brazil, microscopic diagnosis is available in most of the areas with endemic malaria transmission. In the Amazon Region, there are 3,240 active microscopy laboratories (data from January 2008) distributed in all nine states of the Region [9]. However, limitations for diagnosis in some areas persist, which are associated with difficulty in access to services, population mobility and high cost of microscopy in remote locations [10].

In the 1990 s, rapid immunochromatographic diagnostic tests (RDT) were developed. RDT detects specific *Plasmodium *antigens in blood collected from a finger stick. Diagnosis using RDT can be completed in 15 minutes by individuals with minimal training, and its use do not require electricity or any specialized equipment [11]. It offers a useful alternative particularly in remote locations where it is difficult to establish and maintain microscopy laboratories. Disadvantages of the RDT include the inability to identify mixed *Plasmodium *infections, to differentiate between the various *Plasmodium *species, and its high cost [11].

Despite its costs, in areas with limited structure in place for microscopy diagnosis, RDT has been demonstrated to be effective [11] and its use has been recommended by the WHO for endemic remote areas with difficult access [11].

Various studies have reported the high accuracy of the OptiMAL^® ^RDT in Brazil, Colombia, Africa and Asia [12-16]. In Brazil, the National Malaria Control Programme of the Brazilian Ministry of Health recommends RDT use in no endemic areas or in areas of difficult access [17], as laboratory structure for microscopy is not always available in these areas. However, evaluation of costs and cost-effectiveness of its use has not been yet conducted.

The aims of this study were to estimate the total cost of diagnosis of new malaria cases and the cost-effectiveness ratio of using OptiMAL^® ^RDT compared to conventional thick smear microscopy for malaria diagnosis in remote areas of the Amazon Region. This will provide important information to assist national decision makers on the impact of RDT and to formulate public health policies regarding the use of RDT for malaria diagnosis in such areas.

## Methods

### Decision analytic model

**Figure 1:**
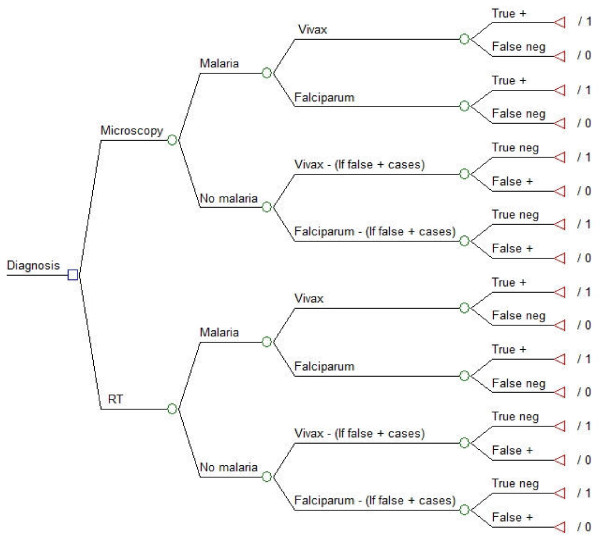
**Decision tree for the "adequately diagnosed cases"**. Notes: RT - Rapid Test; Neg - negative; + - positive;/1 - adequately diagnosed case;/0 - inadequate diagnosis

A decision tree was developed to compare the use of OptiMAL^® ^RDT to the conventional thick smear microscopy as diagnostic strategies for malaria in remote endemic areas (Figure 1).

A hypothetical cohort of all individuals with fever who had a diagnostic procedure for malaria conducted in 2006 (n = 33,491) was simulated considering its various probability nodes. All individuals (100%) presenting with fever to health facilities would undergo diagnostic test using either microscopy or RDT. They could either have malaria or not (prevalence of malaria in the population). If this patient had malaria, the diagnostic test could result positive for malaria (sensitivity) - indicating infection due to *Plasmodium falciparum *or *P. vivax *- representing a true positive result; or result negative (1-sensitivity) representing a false negative result. If this patient did not have malaria, the diagnostic test could result negative (specificity) representing a true negative result; or result positive (1-specificity) representing a false positive result. While true positive and true negative results were considered as adequately diagnosed cases, false negatives and false positives were considered as incorrectly diagnoses cases. These were the terminal nodes of the decision tree (Figure 1). Cost data and epidemiological were collected and inputted to populate this decision tree.

### Study area

Data was collected from remote areas in 12 municipalities in the state of Pará in the Amazon Region, where malaria is endemic. In these municipalities, approximately 30% of febrile cases tested for malaria each year live in areas where no laboratory facility for microscopy diagnosis is available. Risk of malaria considering API in the municipalities varied from 6.28 to 184.27 cases/per 1,000 population in 2006.

In 2006, these municipalities introduced Optimal^® ^RDT for varying periods of time. Thus, some primary data from areas using OptiMAL^® ^RDT was available and collected for this analysis.

In remote areas, malaria diagnosis requires healthcare workers to actively seek out febrile patients and collect their blood. Microscopy diagnosis rely blood collection during the first patient encounter and transportation of this sample to the nearest laboratory where trained technicians can perform diagnosis. Healthcare workers then need to return to the areas to provide the diagnosis to the patient, and therapy for malaria cases is instituted at that time. RDT diagnosis is done on site during the first encounter of healthcare workers with the febrile patient, when finger stick blood is collected. Therapy for malaria positive cases is delivered on site during this same visit as diagnostic results are readily available.

This cost effectiveness analysis took into consideration the above description of provision of diagnosis and care for febrile cases in the area studied. A hypothetical cohort of cases was followed up from fever onset to diagnostic results provided (analytical framework). The study period was January-December 2006. The analysis perspective was that of the Brazilian Public Health System. The outcome considered in the analysis was adequately diagnosed cases of malaria.

### Costs of malaria diagnosis

Direct medical and non-medical costs were considered. All costs were calculated in Brazilian Reais and converted to U.S dollars considering the official average exchange rate for 2006 (US$1.00 = R$2.17) [18].

Costs data was obtained from various sources. Primary data sources was collected at the Municipal and State Health Departments of Novo Repartimento municipality and the state of Pará. Secondary data sources included the scientific literature, official government reports, manuals, and administrative acts from the Malaria Control Programme, the National Strategic Inputs System (SIES), the reimbursement table for procedures by the National Health System (SUS) and the SUS Hospital Information System [19-23].

Costs considered for microscopy diagnostic strategy included: thick smear microscopy with consumables and supplies, equipment costs (microscope purchase and maintenance), transportation for sample collection and delivery of results, and training for healthcare professionals performing thick smear microscopy.

Costs considered for OptiMal^® ^diagnostic strategy included consumables and supplies (gloves and OptiMal^® ^RDT), transportation for sample collection and delivery of results, and training for healthcare professionals performing rapid tests.

### Thick smear microscopy and OptiMal^® ^RDT costs

Consumables and supply costs for microscopy were aggregated into one estimated cost measure of conducting a single thick smear procedure. In the base case, cost of one thick smear procedure was the one estimated by Macauley in Brazil [24] based on costs of all used microscopy supplies through a passive case detection diagnosis strategy. Upper level variation for sensitivity analysis also considered Macauley [24] estimates through a passive plus active case detection diagnosis strategy. As these costs were estimated for 2001, they were adjusted for inflation considering Index Consumers Price [25]. Lower level variations around the base-case were estimated for sensitivity analysis using micro-costing techniques in which the cost of individual consumables required to perform one thick smear procedure were estimated. Primary cost data was obtained from the Malaria Laboratory of the Evandro Chagas Institute of the Brazilian Health Ministry (personal communication). The cost of purchase of the OptiMal^® ^RDT was obtained from the Brazilian Health Ministry [19].

### Equipment costs

The cost of purchase of the microscope was obtained from the Brazilian Health Ministry. It was assumed that there was one microscope available for use for every professional and an annual maintenance for each equipment. The cost of the microscope was averaged over the year based on a discount rate of 5% and an average life of 15 years [26,27].

### Transportation costs

Transportation costs considered for the microscopy diagnostic strategy included two trips of one healthcare professional into the remote area; one for specimen collection and a second one for delivery of results. Transportation costs considered for the RDT considered one trip of one healthcare professional into the remote area for both collection of sample and delivery of results. Transportation costs to remote areas were estimated considering data provided by municipal level on the use of fuel (personal communication). The prices of gasoline and diesel for 2006 were collected from the official Brazilian government website [28,29].

### Training costs

Training costs considered one annual 40-hour training for the thick smear microscopy procedure and one annual 150 minutes training for the RDT [30]. The estimated cost was obtained from the Brazilian Health Ministry considering the microscopy training held in 2006.

Costs of equipment, transportation, and salary staff which are shared with other health programs were estimated considering WHO's parameters which determine the number of microscopy diagnostic tests performed per hour at four different levels of malaria prevalence [31]. Thus, the study area was stratified according to API into four prevalence categories and the number of healthcare professionals needed to conduct the diagnostic procedures was estimated. Costs of equipment, transportation, and salary staff where then estimated, assuming them to be directly proportional to the number of healthcare professionals needed to conduct the diagnostic procedures. The total for each diagnostic strategy for these items represented the average costs in the study area weighted by prevalence in each risk strata.

Costs of quality control procedures for microscopy and RDT diagnostic methods and costs of construction and maintenance of laboratories were not considered. Table 1 presents cost components and their respective unit costs for microscopy and OptiMal^® ^RDT diagnosis.

**Table 1:** Cost components and unit costs considered for malaria diagnosis, Amazon Region, Brazil in 2006.

Items	Unit cost considered in the base-case analysis and variation (US$)	Information sources
**Exams and supplies**		
Thick smear - one exam ^(1)^	0.92 (0.19-1.40)	Base-case: Macauley, 2005
		Variation: Malaria Lab of Evandro Chagas Institute; Macauley,2005
OptiMal^® ^- one test	4.28 (3.59	Base-case: Union Official Gazette, 2006
	- 4.93)	Variation: DiaMed Laboratory; State of Pará
Gloves to use with RDT	0.02 (0.05)	Base-case and variation: Stock Prices of Ministry of Health

**Salaries**		
Microscope technician - monthly salary	290.93	Base-case: Health care center/Municipality Novo Repartimento
Health worker - monthly salary	214.38	Base-case: Health care center/Municipality Novo Repartimento

**Equipment**		
Microscope - one unit - annual value	485.65 (327.94)	Base-case and variation: Ministry of Health
Microscope maintenance - one annual maintenance	36.87	Base-case: Malaria Lab of Evandro Chagas Institute

**Transportation**		
For the rapid test - average monthly cost	1,450.23 (855.30 - 16,428.14)	Base-case Health care center/Municipality Novo Repartimento
For microscopy - average monthly cost	2,900.46 (1,710.60 - 32,856.28)	Base-case and variation: Health care center/Municipality Novo Repartimento

**Training**		
Microscopy - one annual course per municipality	11,137.79 (5,934.34)	Base-case and variation: Ministry of Health
Rapid test - one annual course per municipality	116.02 (61.82)	Base-case and variation: Ministry of Health

Notes:

Corresponds to the individual cost of an examination, which includes a glass slide, Giemsa and other stains (all components of stains), oil immersion, lancet, cotton, alcohol, and gloves.

### Epidemiological parameters

Secondary sources of epidemiologic data were considered and obtained from the scientific literature (medline, Lilacs and SciElo databases), and the National Malaria Surveillance Information System of Health Ministry [9].

These data included prevalence of malaria, proportion of malaria cases due to *P. vivax *and *P. falciparum *species, sensitivity and specificity of microscopy and OptiMal^® ^RDT for both *P. vivax *and *P. falciparum *infections (Table 2).

**Table 2:** Epidemiologic parameters considered in the analytic model, Amazon Region, Brazil, 2006.

PARÂMETER	BASE-CASE VALUE	VARIATION VALUES	INFORMATION SOURCES*
Prevalence of malaria among febrile patients seeking diagnosis ^(1)^	24.6%	12.7% - 28.5%	Base-case and variation: SIVEP/Malaria

Proportion of malaria cases due to *Plasmodium vivax *^(1) ^	67.2%	63.6% - 92.4%	Base-case and variation: SIVEP/Malaria

Proportion of malaria cases due to *Plasmodium falciparum *^(1)^	32.8%	7.6% - 36.4%	Base-case and variation: SIVEP/Malaria

Sensitivity of microscopy for *Plasmodium vivax*	95%	77% - 82%	Base-case: Ohrt et al., 2002
			Variation: Haghdoost et al.,2006; Iqbal et al., 1999

Specificity of microscopy for *Plasmodium vivax*	100%	95.0% -100.0%	Base-case: Haghdoost et al.,2006
			Variation: Haghdoost et al.,2006

Sensitivity of microscopy for *Plasmodium falciparum*	92%	83% - 92%	Base-case: Ohrt et al., 2002
			Variation: Humar et al., 1997

Specificity of microscopy for *Plasmodium falciparum*	100%	99% - 100%	Base-case: Ndao et al., 2004
			variation: Humar et al., 1997

Sensitivity of OptiMal^® ^for *Plasmodium vivax*	92%	62.5% -100%	Base-case: PAHO Report, elaborated by Fontes, 2002
			Variation: Ratsimbasoa et al.,2007; Londoño et al., 2002

Specificity of OptiMal^® ^for *Plasmodium vivax*	100%	80% - 100%	Base-case: PAHO Report, elaborated by Fontes, 2002
			Variation: Londoño et al., 2002

Sensitivity of OptiMal^® ^for *Plasmodium falciparum*	95.6%	62.3% - 98.8%	Base-case: PAHO Report, elaborated by Fontes, 2002
			Variation: Palmer et al., 1998; OPAS Report, elaborated by Fontes, 2002

Specificity of OptiMal^® ^for *Plasmodium falciparum*	99.6%	89.3% - 100%	Base-case: PAHO Report, elaborated by Fontes, 2002
			Variation: Ferro et al.,2002; Ratsimbasoa et al.,2007

Notes: * To the "Variation values", the first citation refers to the lowest value,

and the second citation refers to the highest value.

^(1) ^The base-case values correspond to the values aggregated from 12 municipalities, and variation values correspond to the lowest and highest values found in the different risk strata presented in the 12 municipalities.

Accuracy studies for OptiMAL^® ^RDT were considered only if they used microscopy as the gold standard in endemic areas. Quality of published studies was assessed considering 14 criteria of the *Quality Assessment of Diagnostic Accuracy Studies (QUADAS) *instrument [32] and three additional criteria judged relevant: socio-demographic characteristics of patients, confidence interval and the sampling method as proposed by the *Standards for Reporting Studies of Diagnostic Accuracy (STARD) *[33]. From 30 studies, only 14 fulfilled minimum quality criteria. From these, five studies were deemed as being of better quality considering the above criteria [7,12-15]. These were the sources of the sensitivity and specificity values in the case base and variation considered in sensitivity analysis. Accuracy studies of thick smear microscopy were considered only if they used polymerase chain reaction as the gold standard [6,16,34-36].

### Cost, cost-effectiveness and sensitivity analysis

*TreeAge Pro^® ^*software was used to build the decision model and for cost-effectiveness and sensitivity analyses [37]. Total costs accrued for malaria diagnosis during the study period were estimated for both diagnostic strategies considering the total number of exams performed during the study period (n = 33,491). Cost per adequate diagnosis was obtained for both microscopy and OptiMAL^® ^RDT diagnostic methods by dividing total costs by the estimated number of cases diagnosed.

The cost-effectiveness decision model predicts the number of adequate diagnosis of febrile patients with suspected malaria and costs associated with each diagnosis. Incremental cost-effectiveness ratio (ICER) was calculated considering the incremental cost needed to adequately diagnose one individual with suspected malaria using OptiMAL^® ^RDT as opposed to microscopy.

In order to examine the variability of the cost-effectiveness ratios, one-way sensitivity analysis to investigate the effect of key parameter values was conducted. Cost parameters for microscopy and RDT diagnosis (exams and supplies, equipment, transportation and training) were varied, as were also malaria prevalence and accuracy estimates for both diagnostic methods. Parameter values varied over the upper and lower range estimates.

Variation of costs (Table 1) and epidemiologic parameters (Table 2) considered in the analysis and their respective sources of information are presented.

## Results

Considering 33,491 diagnostic procedures for malaria conducted in 2006, the total cost of the microscopy strategy would be US$227,315.53, and for the OptiMal^® ^RDT, US$172,082.09 (Table 3). The cost per adequately diagnosed case was US$ 5.14 using OptiMal^® ^RDT and US$ 6.79 using microscopy (Table 4).

**Table 3:** Total costs of diagnostic strategies for 33,491 exams performed in the study area, Amazon Region, Brazil, 2006.

Items	Microscopy strategy (US$)	RDT strategy (US$)
Thick smear microscopy	30,712.94	**--**
OptiMal^® ^rapid test	**--**	143,224.18
Latex gloves to use with RDT	**--**	771.68
Salary of technician to read smear using microscopy	23,826.86	--
Salary of health workers	17,557.55	17,557.55
Microscopes	3,059.56	**--**
Maintenance of microscopes	232.26	**--**
Transportation	18,272.90	9,136.45
Training	133,653.46	1,392.22

**Cost of diagnosis**	**227,315.53**	**172,082.09**

**Table 4:** Results of the cost-effectiveness analysis of the microscopy compared to OptiMal^® ^, Amazon Region, Brazil, 2006.

Strategy	Cost per case (US$)	Additional cost (US$)	Effect	Additional effect	ICER (US$)
OptiMal^®^	5.14		0.982		
Microscopy	6.79	1.65	0.985	0.003	**549.92**

Considering the total 33,491 tests performed, microscopy and RDT adequately diagnosed 32,989 and 32,888 cases, respectively. In the base-case scenario, RDT was less effective failing to diagnose 3 cases in 1,000 febrile patients comparing to microscopy. Thus, incremental cost-effectiveness ratio was US$ 549.92 per adequately diagnosed case for microscopy when compared to OptiMal^® ^RDT (Table 4). Results are presented in reverse, to avoid negative values, so actually is presented CE ratios of microscopy compared to RDT.

### Sensitivity analysis

In sensitivity analysis, cost-effectiveness ratios were sensitive to some parameters (Table 5). The largest impact on results were observed when sensitivity of microscopy for both Plasmodium species was lower than base-case, resulting in microscopy being both more costly and less effective and therefore dominated by the RDT strategy. This was observed when microscopy sensitivity for *P. vivax *was 0.90 or lower, sensitivity for *P. falciparum *0.83 or lower, and microscopy specificity for *P. vivax *was 0.98 or lower.

**Table 5:** Results of the sensitivity analysis, Amazon Region, Brazil, 2006.

Parameter and variation value *	Incremental cost-effectiveness ratio (ICER) (US$)
Microscopy sensitivity for *P. vivax*	
0.90	Microscopy dominated
Microscopy specificity for *P. vivax*	
0.98	Microscopy dominated
Microscopy sensitivity for *P. falciparum*	
0.90	1,649.77
0.83	
	Microscopy dominated
Specificity of OptiMal^® ^kit for *P. vivax*	
0.95	58.92
0.80	15.86
Cost of OptiMal^®^	
4.93	333.33
3.59	778.80
Cost of transportation to perform one rapid test	
3.08	Rapid test dominated
0.16	586.79
Cost of transportation to perform one thick smear	
6.18	2,428.57
2.12	Rapid test dominated

Cost-effectiveness analysis of the microscopy compared to OptiMal^®^.

Note: * Only variation values in which changes in ICER occurred are presented.

When specificity of OptiMal^® ^RDT was lower, cost-effectiveness ratio changed proportionately, with an incremental cost-effectiveness varying from US$ 15.86 - 58.92 for each additional adequate diagnosis using microscopy. When higher transportation costs to perform RDT were assumed, RDT was dominated by microscopy. Changes in the other parameters did not result in changes in the cost-effectiveness ratios.

## Discussion

Although OptiMal^® ^RDT is more expensive than thick smear when only the procedure costs are considered, this study demonstrated that when all cost components of malaria diagnosis are considered - personnel, equipment, maintenance, training and transportation - microscopy is more costly than OptiMal^® ^RDT. In remote areas, routine malaria diagnosis requires active case search by community health agents, and at least two trips in order to collect specimens and provide diagnoses to suspected cases.

Microscopy strategy was more effective and more cost-effective than OptiMal^® ^in remote endemic areas when high sensitivity (92-95%) and specificity (100%) were maintained.

The model was sensitive to small variations of specimen-specific sensitivity and specificity of microscopy for malaria diagnosis. When sensitivity and specificity of microscopy for *P. vivax *were 0.90 and 0.98, respectively, and when its sensitivity for *P. falciparum *was 0.83, OptiMal^® ^RDT was more cost-effective than microscopy, indicating that very high microscopy accuracy levels are needed for microscopy to be more cost-effective than RDT. Unlike newly available technologies, when the price of one RDT OptiMal^® ^test was reduced, results did not vary significantly.

Lubell *et al *[38], Shillcutt *et al *[39], Rolland *et al *[40], and Chanda *et al *[41] prompted the need for alternative malaria diagnostic methods in studies conducted in Africa, where low accuracy of diagnosis was observed. In countries where presumptive malaria treatment considering clinical signs and symptoms is recommended, specificity of diagnosis strategy is close to zero, and the use of RDT for malaria diagnosis was demonstrated to be very cost-effective when compared to standard practice as this would avoid presumptive treatment of false positive cases [39,40]. Lubell *et al *[38] and Chanda *et al *[41] demonstrated that RDT was cost effective when compared to microscopy also in settings where microscopy accuracy was low.

Only one published study was conducted in the Amazon Region, which is endemic for malaria [42]. It was demonstrated that in remote areas RDT led to cost-savings for outcomes considered - timely and appropriate treatment, serious malaria cases and deaths averted - when compared to microscopy. However, this study did not consider diagnostic method accuracy as parameters in the model, and therefore it is difficult to compare these results. A cost-minimization analysis conducted in a rural locality in Brazil demonstrated that the RDT ParaSight-F^® ^was more cost effective than microscopy, mainly due to significantly lower transportation costs when using RDT [43].

This study demonstrated that microscopy was cost-effective when compared to OptiMal^® ^when high accuracy of thick smear technique was maintained.

True accuracy of microscopy diagnostic techniques in the field is unknown in most endemic countries. This is due to difficulties in establishing adequate gold standard against which to determine sensitivity and specificity of microscopy.

In the Brazilian Amazon Region, the infra-structure for microscopy diagnosis is in place in most areas and, therefore, the study did not considered the capital costs of setting up this structure, which is known to be costly. If considered, microscopy costs would be higher. Therefore, RDT use may be the best available diagnostic alternative in remote endemic areas where infra-structure for microscopy diagnosis is not in place, or where high accuracy of microscopy cannot be assured.

The model was also sensitive to variations in OptiMal^® ^specificity to *P. vivax*, leading to lower cost per each additional diagnosed case for microscopy when OptiMal^® ^specificity was lower. Although various different RDTs are available, they differ significantly in sensitivity and specificity values. When making informed decisions on RDT introduction, a product with high accuracy should be selected, as recommended by the WHO [11].

Finally, further developments on determining gold standards and processes to evaluate sensitivity and specificity of microscopy diagnostic procedure for malaria are urgently needed. Decision making on the use of the microscopy or RDT as alternative diagnostic procedure in remote endemic areas considering cost-effectiveness is strongly dependent upon knowledge of true accuracy of microsocopy in the field.

## Conclusion

Microscopy is more cost-effective than OptiMal^® ^in these remote areas if high accuracy of microscopy is maintained in the field. Decision regarding use of rapid tests for diagnosis of malaria in these areas depends on current microscopy accuracy in the field.

## Competing interests

The authors declare that they have no competing interests.

## Authors' contributions

MRFO conceived the study, participated in the design of the study, performed the analysis and drafted the manuscript. ACG conceived the study and participated in its design and coordination. CMT conceived the study and participated in the design and analysis of the study. All authors read and approved the final manuscript.
